# Effect of myrtle leaves integration in sheep diet and its addition as powder on leg meat' oxidative stability, physicochemical, microbiological and sensory properties during storage

**DOI:** 10.1002/fsn3.3219

**Published:** 2023-01-06

**Authors:** Souha Tibaoui, Ines Essid, Samir Smeti, Naziha Atti

**Affiliations:** ^1^ Laboratoire de Productions Animales et Fourragères, INRA‐Tunisia University of Carthage Ariana Tunisia; ^2^ UR‐UR PATIO (UR17AGR01), INAT University of Carthage Tunis Tunisia

**Keywords:** antioxidants, cull ewes, distillate myrtle leaves, leg meat, lipid oxidation, microbiological quality

## Abstract

The synthetic antioxidant improved the shelf life of meat products; however, consumers start to reject them for health reason. For this, the natural antioxidants like Myrtle distillate leaves (MDL) could be an interesting alternative and potential natural antioxidant given their richness in bioactive compounds. This study aimed to test the effect of myrtle distillate leaves (MDL) as natural antioxidant in premortem phase as diet of cull ewes and in postmortem phase in form of powder added to deboned legs on meat' quality. All ewes received individually 500 g of hay and 750 g of concentrate for Control (C) group; for Myrt group, the concentrate was partially replaced (400 g) by pellets containing 30% MDL and 350 g concentrate. For each ewe, both legs were used for the experiment; one leg was treated with MDL powder and the other with Control. Meat from ewes' fed MDL presented better scores for red color, aftertaste and juiciness, than control group. Meat treated with MDL powder showed the highest values of total phenolic, α‐tocopherol content and redness score with lower lipid oxidation (*p* < .05). The microbiological quality of meat was not affected by MDL. Myrtle distillate leaves could be used in different forms, in order to obtain higher meat product quality.

## Graphical abstract

The article showed that distille myrtle leaves can be potentially used as natural antioxidant in meat product preservation.

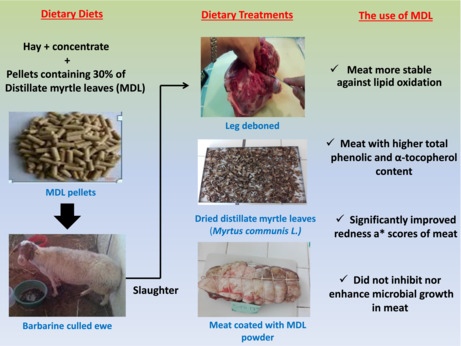

## 1 INTRODUCTION

Changes in lifestyle and eating habits, has been shown as the main cause of diseases such as obesity, anemia, and cardio vascular diseases. Consumers are becoming more health‐conscious and tend to search foods which contain bioactive component and polyunsaturated fatty acids (PUFA), considered beneficial for human health. Among food, meat and meat products are the most prone to bacterial spoilage and lipid oxidation that occured during the processing and storage and caused a decrease in their quality (Mielnik et al., 2008). Oxidative deterioration is an important matter of concern to industrials because it affects the nutritional value of the product by decomposition of vitamins and PUFA; furthermore, it leads to discoloration and to the development of off‐flavor which strongly affect meat's shelf life (Allen & Cornforth, 2010). In addition, microbial deterioration also plays a major role in quality deterioration. The animal's dietary background was also a major varying source of healthy PUFA, especially omega‐3 PUFA, and antioxidant factors in sheep meat (Atti et al., 2013; Ben Abdelmalek et al., 2020). Thus, having stable meat products requires the use of conventional and synthetic additives. However, consumers' concerns rise about the safety of such synthetic substances and their potential health risks which led to a growing tendency toward the use of natural antioxidant as alternative in food preservation (Stefanello et al., 2015). Natural additives derived from plant' extracts, culinary herbs, and vegetable products and residual sources from industry of aromatic plant' distillation rich in polyphenols representing the major active components responsible for the antioxidant activity (Ahn et al., 2007). In this regard, some of these residuals, such as rosemary and thyme distilled leaves, have been the subject of many investigations and their richness in phenolic antioxidants was shown (Ben Abdelmalek et al., 2020; Yagoubi et al., 2021). Moreover, several authors reported the antioxidant effect of plant extracts such as green tea extract (Bozkurt, 2006), apple peel (Wolfe & Liu, 2003); the rosemary residues antimicrobial activity was also documented (Ben Abdelmalek et al., 2018; Essid et al., 2018). Hence, the addition of these natural extract to raw and cooked meat products was proven to have decreased lipid oxidation, improved color stability and total antioxidant capacities which are important characteristics for shelf stable meat products (Ahn et al., 2007; Smeti et al., 2021). The myrtle (*Myrtus communis* L.), an aromatic plant shrub disseminated throughout the Mediterranean area and widely used in folk human‐medicinal practices as an antimicrobial and antioxidant (Aidi‐Wannes et al., 2010), was rarely used as a natural antioxidant and antimicrobial in meat and meat products (Manzoor et al., 2014). On the other hand, culled ewes are generally characterized by poor body condition; meat is known for its low quality attributes (Ben Abdelmalek et al., 2020) and merits better valorization. Therefore, the present study aimed to valorize culled ewes' leg meat added with distillate myrtle residues as natural antioxidant used in premortem and in postmortem (as distillate myrtle leaves' powder) on lipid oxidation, color deterioration, and microbial quality.

## 2 MATERIALS AND METHODS

All procedures employed in this study (transport and slaughtering) met the ethical guidelines and adhered to Tunisian legal requirements (The Livestock Law No. 2005‐95 of 18 October 2005, Chapter II; Section 1 and Section 2 relative to the slaughter of animals).

### 2.1 Distillate myrtle leaves preparation

The myrtle distillate leaves (MDL) were generated after myrtle' essential oils extraction by hydro‐distillation. They were collected from a distillation unit in the Northwest of Tunisia (Nefza). In this experiment, they were applied to sheep meat during the premortem and postmortem phases. For culled ewes' feeding, the MDL were air‐dried in the shade for one week to ensure complete dehydration. Then, dried MDL were ground and mixed in the manufactory with other ingredients to formulate pellets containing 30% MDL, 12% soya‐bean, and 58% barley. For postmortem use, the MDL were washed, and dried for 12 h at 60°C using an electric food dehydrator to ensure complete dehydration. Dried leaves were then ground to fine powder using laboratory grinder in order to obtain the MDL powder. The total polyphenol content was 153 mg TAE/g for MDL powder.

### 2.2 Processing of culled ewes' meat product

In the premortem treatment eighteen Barbarine culled ewes (5–6 years old) were used in a 90‐day feeding trial. They were divided into two groups of nine, reared in individual boxes and fed with 500 g of hay and 750 g of concentrate for control (C) group, 500 g of hay, 350 g of concentrate, and 400 g of MDL pellets for the second group (Myrt). For this group, the concentrate was partially substituted by pellets containing 30% of MDL. The total polyphenol content was 3.07 and 25.17 mg TAE/g for concentrate and Myrt‐pellets, respectively. At the end of fattening period ewes were slaughtered, after cooling during 24 h at +4°C, the cold carcasses were cut into pieces and all legs were conserved for this study. For the postmortem treatment, in both groups (Myrt and C), both legs were deboned from each animal. The meat of one leg (M) was completely coated with MDL powder (3 g of MP/kg of meat); the second leg, served as control (C) with no additive, to obtain a total of 18 legs meat per group (9 treated with MDL powder and 9 served as control). Therefore, we obtained four groups with 9 samples each: CC, CM, MyrtC and MyrtM. Each leg' meat was then stringed and stored in a permeable plastic box at 4°C for 6 days. The physico‐chemical and microbiological parameters were measured on days 0, 2, 4, and 6 of storage, while sensory evaluation and antioxidant activity were evaluated on day 4 of storage. All measurements were replicated thrice.

### 2.3 Meat's total phenolic content (TPC)

The TPC of each leg meat was measured, in triplicate, according to the Folin–Ciocalteu method of Vázquez et al. (2015). Briefly, a mixture of 9 ml milli‐Q water, 10 ml aqueous solution of methanol (50/50; v/v), 500 μl of Carrez I solution and 500 μl of Carrez II solution were added to 1 g of ground meat After vortexing for 1 min, 5 ml of acetonitrile was added and dissolved. The mixture was homogenized and centrifuged at 2264 *g* for15 min at 4°C, the supernatant was filtered through a 0.22 μm PTFE filter, and the extract obtained was used to determine TPC. To 15 μl of extract, 147 μl of water milli‐Q, 13 μl of Folin–Ciocalteu reagent, and 7% Na_2_CO_3_ were added. The samples were left at room temperature for 1.5 h in the dark, after which, the absorbance was measured with a spectrophotometer (Thermo Electron Corporation) at 750 nm and the results were expressed as mg tannic acid equivalents (TAE)/g dried sample.

### 2.4 Meat's vitamin E content

**FIGURE 1:**
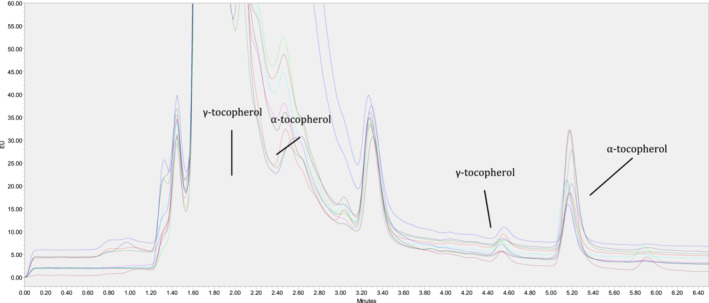
All chromatograms with rescaled (fluorescence signal and time)

The Vitamin E evaluation was assayed by UHPLC as described by Prates et al. (2006). To 0.2 g of lyophilized samples, 0.2 g of ascorbic acid and 3 ml of saponification solution were added, the mix was transferred to glass tube of 25 ml and mixed with 10% w/v of KOH with 50% EtOH and 50% of distilled water. The tubes were vortexed under nitrogen atmosphere, and left overnight in an orbital shaker, then 5 ml of hexane‐ethyl acetate (9:1 v/v) and 5 μg/ml of BHT (purity 99.9% GC, Sigma Aldrich) were added. The mixture was vortexed and centrifuged for 5 min at 1370 *g* and 10°C. The supernatant was recovered, transferred into glass tube of 10 ml, and then evaporated using a rotational vacuum concentrator (Christ RVC2‐25) for 45 min at 40°C. The residue obtained was resuspended in 1 ml of mobile phase acetonitrile: methanol: dichlorometane (75:15:10, v:v:v), and the mixture was vortexed and shacked in an orbital shaker for 10 min and the aliquot of n‐hexane layer was filtered through a 13 mm × 0.20 μm PTFE filter into an amber screw‐cap vial of 2 ml. The chromatographic system used was an Acquity UPLC H‐Class liquid chromatograph (Waters) equipped with fluorescence (Waters 2475 Multi Fluorescence Detector) and absorbance (PDA eλ Detector) detectors, and an Acquity UPLC HSS T3 column 2.1 mm × 15 mm × 1.8 μm (Waters). Tocopherols were detected by fluorescence emission at 295 nm excitation wavelength (λexc) and 330 nm emission wavelengths (λemi) as shown in the chromatograms (Figure 1). To determine cholesterol concentration, the same extraction method for Vitamin E was used and it was detected by absorbance at 220 nm. The analytes in all samples were identified by comparison of the retention times and spectral analysis with those of the pure standards.

### 2.5 Meat's antioxidant activity (ABTS assay)

The extraction process was based on the protocol of Saura‐Calixto and Goñi (2006) for ABTS 2,2′‐azino‐bis (3‐ethylbenzothiazoline‐6‐sulfonic acid) assay and DPPH (2,2‐diphenyl‐1‐picryl‐hydrazyl‐hydrate) radical scavenging activity. The ABTS radical cations (ABTS+) were prepared by reacting a 7.4 mM ABTS stock solution with 2.45 mM potassium presulfate (1:1, v/v). The stock solution was kept in the dark at room temperature for 12–16 h. The radicals were stable in this for 2 days when stored under these conditions, and then the stock solution was diluted with ethanol to obtain absorbance of 0.7 ± 0.02 at 734 nm. For spectrophotometric assay, 20 μl aliquot of the sample was added to 280 μl of ABTS solution and placed in the dark for 30 min. Methanolic solutions of known trolox concentrations were used for calibration. Absorbance was recorded at 730 nm and results were expressed as mg trolox equivalents (TE)/g dried sample.

### 2.6 Color parameters and pH of leg meat during storage

The color changes in leg meat during storage were monitored with a portable Chromameter (CM‐2006 d; Konica Minolta Holdings, Inc.). The measurements were performed directly on meat's surface with a measured area of 8 mm, standard illuminant D65, and an observer angle of 10° and expressed with L* (lightness), a* (redness), b* (yellowness) and C (chroma), scale values of the CIELAB system (CIE, 1976). The pH of leg meat was measured throughout the storage period with a penetrating electrode connected to a pH meter (HI 99163; Hanna Instruments) and calibrated with two buffers (7.00 and 4.01) at 25°C. For each sample, all measurements were done in triplicate.

### 2.7 Lipid oxidation (TBARS)

Lipid oxidation was determined during the storage period, by measuring thiobarbituric acid reacted substances (TBARS) values according to Botsoglou et al. (1994) method, with modifications. Briefly, 10 g of minced meat was homogenized with 20 ml of 10% trichloro acetic acid using an Ultra‐Turrax (T25; IKA‐Labortechnik) for 10 s at 20,376 *g*. The mixture was centrifuged for 30 min and the supernatant was recovered then decanted through a paper filter and mixed with thiobarbituric acid solution. The tubes were incubated at 97°C in a water bath for 15 min to develop a pink chromogen. After cooling, the absorbance of sample was recorded at 532 nm by a spectrophotometer (Thermo Electron Corporation). A standard curve was prepared using 1,1,3,3‐tetramethoxypropane (99%) with increasing concentrations (from 0 to 120 μl), 5 ml of thiobarbituric acid and 5 ml of water. TBARS values were calculated and expressed as mg MDA equivalents/kg.

### 2.8 Microbial analysis

For microbiological measurements, 10 g of ground meat sample was aseptically weighed and homogenized with 90 ml of sterilized peptone water using Clear Line stomacher blender bags (Clear Line), for about 2 min at room temperature. Serial 10‐fold dilutions were prepared using sterile peptone water as diluent, then aliquots of the appropriate dilutions were spread or poured in different selective agars used according to ISO 6887‐2 (2004). Total Viable Counts (TVC) were enumerated on Plate Count Agar (PCA) and incubated at 30°C for 24–48 h. Sabouraud agar was used for yeasts and molds after incubation at 25°C for 2–3 days. The quantification of lactic acid bacteria (LAB) was determined by spread plating and counting on MRS agar at 30°C for 48 h. Fecal and total coliforms were cultivated on Desoxycholate agar (0.1%) after 48 h at 44°C and for 24 h at 35°C, respectively. Baird Parker Medium agar used for *Staphylococcus aureus* enumeration following incubation at 37°C for 24 h. The tryptone‐sulfite‐neomycin (TSN) agar was used for the enumeration of *Clostridium perfringens*. All analyses were performed in triplicate, for each leg, and the counts were expressed as log10 of colony forming units/g (log CFU) for each sample.

### 2.9 Sensory evaluation

#### 2.9.1 Samples' preparation

For each sensory evaluation session, four samples of leg meat (one for each group) were thawed at a temperature of 4°C for a period of 24 h. Meat samples were wrapped in aluminum foil and baked in an electric oven at 180°C for about 40 min, to an internal temperature of 65°C. The meat was allowed to rest for 5 min, in which time an endpoint temperature of 72°C was reached (AMSA, 1995). Immediately after cooking, leg meat, from each group, was divided in 2 cm × 2 cm × 0.5 cm samples, wrapped in aluminum foil, placed in a preheated oven at 60°C, and evaluated within 10 min.

#### 2.9.2 Sensory analysis

An analytical sensory evaluation panel consisting of fifteen trained members, from the laboratory staff, was used to evaluate the meat. Descriptive analysis was carried out to evaluate the intensities of sensory characteristics. Panelists were asked to rate each sample for the sensory attributes: color, odor, flavor, texture, juiciness (water perceived during mastication), firmness (the force needed to chew), cohesiveness and overall acceptability and indicate a point on a nine‐point hedonic scale corresponding to the intensity of their different feelings for each attribute as follow: 1 = very low intensity, 3 = low intensity, 5 = medium intensity, 7 = intensive and 9 = very intensive. The sensory evaluation consisted of 5 sessions. For the first four sessions, 8 different leg meat samples were divided into two sets of four (one for each group), and immediately after cooking, samples from the first set were presented for assessors, in a random order, on a white dish and coded with a randomly selected 3‐digit number. Approximately 2 min elapsed, before evaluating the second set. For the last session, panelists were asked to evaluate only one set. Bread and water were provided for the trained panelists to cleanse the palate between every two samples (AMSA, 1995).

#### 2.9.3 Statistical analysis

Statistical analyses were performed with SAS (2004). Data of leg meat's antioxidant activity and sensory analysis were tested by analysis of variance using a 2 × 2 factorial with two types of diet (Myrt: group that received MDL in the diet vs. C: control group that did not receive MDL in the diet) and two treatments of leg' meat presence of MDL powder in leg meat or not (C), then with the Tukey's multiple comparison test was used. Data were analyzed according to the following model:

Yijk=μ+Ri+Lj+eijk,

where Yijk represents the responses of samples from ewe k receiving the diet i and treated or not with MDL powder j; μ = mean; Ri = diet (i = ewes received MDL or not); Lj = presence of MDL (j = samples treated with MDL powder or not); eijk = the aleatory error.

For pH, color, TBARS and microbiological measurements during storage, a Linear‐Mixed model analysis for repeated measures was conducted, with the presence of MDL in animal diet, the presence of MDL powder in leg meat and storage time, as the variable factors. The GLM procedure of Statistical Analysis System's Procedures (SAS 2002) was used.

## 3 RESULTS

### 3.1 Leg meat's antioxidant activity

The results of leg meat's antioxidant activity are shown in Table 1. The radical scavenging activity (ABTS) was not affected by MDL intake (*p* > .05) while its addition in deboned leg meat resulted in higher ABTS for MyrtM and CM samples. However, the integration of MDL in the ewe's diet and leg' meat formulation significantly increased the meat α‐tocopherol and TPC content.

**TABLE 1:** Effect of distillate myrtle leaves (MDL) addition in pre and postmortem phases on leg meat antioxidant activity of culled ewes

Diet	Myrt		C		Statistics		
Treatment	MyrtC	MyrtM	CC	CM	*p*‐MDL in Diet	*p*‐MDL powder	MSE
ABTS (mg TE/g)	0.97^b^	1.47^a^	1.08^b^	1.28^ab^	.81	.012	0.11
Polyphenols (mg GAE/g)	85.36^c^	133.81^a^	66.51^d^	106.75^b^	.04	.05	11.48
α‐tocopherol (μg/g DM)	8.43^b^	11.60^a^	4.58^c^	8.88^b^	.01	.011	1.04
γ‐tocopherol (μg/g DM)	0.20^b^	0.23^b^	0.45^a^	0.43^a^	<.01	.003	0.07

*Note*: C: ewes from control group fed oat hay plus concentrate; Myrt: ewes fed MDL pellets; MyrtM: leg' meat from Myrt group added with MDL powder; MyrtC: leg' meat from Myrt group; CC: leg meat from C group; CM: leg' meat from C group added with MDL powder; a. b. c: different letters within the same row (different storage days) differ significantly (*p* < .05); MSE: Standard error of the mean.

### 3.2 Deboned leg meat' color parameters and pH changes during refrigerated storage

The changes in color parameters during storage were presented in Table 2; the interactions were not significant, since they were not reported in the table. The ewe's MDL intake did not affect (*p* > .05) color indices even though a* tended to be higher for meat from Myrt than C group. The MDL powder addition to leg meat did not affect the lightness L*, which was higher than 35 for meat of all treatments. However, it significantly affected (*p* < .05) the redness values a* that decreased for meat of both diet types (C and Myrt) compared to non‐treated legs. Also, the yellowness (b*) was augmented (*p* < .05) by adding MDL powder to leg' meat. During storage, all color parameters significantly (*p* < .05) decreased except the lightness (L*) values. The Redness (a*) decreased throughout storage period for all types of product. However, this decrease was in a slower rate for both treated legs with MDL powder (MyrtM and CM) suggesting that the use of this powder may inhibit the oxidation rate in the product resulting in meat color preservation.

**TABLE 2:** Effect of distillate myrtle leaves (MDL) addition in pre and postmortem phases during storage on leg meat color parameters, pH and TBARS

	Storage days					Statistics			
	Treatment	0	2	4	6	*p*‐MDL in Diet	*p‐*MDL powder	*p*‐Time	MSE
L	CC	39.8 ± 1.66	39.5 + 5.16	42.7 + 2.62	38.5 + 1.07	.92	.82	.35	0.69
	MyrtC	35.3 ± 1.32	37.5 ± 3.38	39.4 + 1.84	44.6 + 1.21				
	CM	41.5 ± 1.88	38.5 ± 1.96	38.6 ± 2.26	37.1 ± 2.22				
	MyrtM	38.9 ± 1.02	36.9 ± 1.29	37.2 ± 0.99	35.4 ± 1.36				
a*	CC	17.8 ± 0.91	13.0 ± 1.62	11.2 ± 0.87	10.1 ± 1.33	.06	.04	<.01	0.33
	MyrtC	18.4 ± 0.47	15.6 ± 0.36	13.5 ± 0.12	10.4 ± 0.62				
	CM	15.7 ± 1.28	10.3 ± 1.21	12.0 ± 1.53	10.2 ± 0.92				
	MyrtM	16.3 ± 0.69	13.1 ± 0.75	13.3 ± 0.87	12.4 ± 0.84				
b*	CC	10.4 ± 0.75	8.3 ± 0.62	3.7 ± 0.74	3.5 ± 0.61	.75	<.01	<.01	0.18
	MyrtC	9.1 ± 0.47	7.2 ± 0.37	5.4 ± 0.66	3.6 ± 0.13				
	CM	13.8 ± 0.80	10.3 ± 0.44	8.7 ± 0.32	5.4 ± 0.72				
	MyrtM	11.0 ± 0.53	10.2 ± 0.53	7.9± 0.63	6.0 ± 0.44				
C	CC	17.9 ± 0.75	14.6 ± 0.87	13.2 ± 1.01	12.2 ± 0.98	.15	.15	<.01	0.25
	MyrtC	19.9 ± 0.80	16.5 ± 0.15	13.3 ± 0.76	12.0 ± 0.56				
	CM	16.9 ± 0.98	15.2 ± 0.82	14.9 ± 0.74	14.0 ± 0.61				
	MyrtM	18.1 ± 0.68	16.9 ± 0.57	14.9 ± 0.89	12.8 ± 0.65				
TBARS (mg of MDA/kg)	CC	0.55 ± 0.05	0.72 ± 0.06	1.01 ± 0.20	1.03 ± 0.20	.04	.03	<.01	0.03
	MyrtC	0.43 ± 0.09	0.52 ± 0.11	0.77 ± 0.11	0.84 ± 0.16				
	CM	0.16 ± 0.06	0.46 ± 0.05	0.47 ± 0.07	0.73 ± 0.07				
	MyrtM	0.28 ± 0.02	0.29 ± 0.02	0.56 ± 0.05	0.81 ± 0.10				
pH	CC	5.93 ± 0.05	6.00 ± 0.06	5.83 ± 0.04	5.94 ± 0.05	.27	.03	.01	0.02
	MyrtC	6.03 ± 0.07	5.88 ± 0.04	5.81 ± 0.02	5.84 ± 0.05				
	CM	5.93 ± 0.05	5.64 ± 0.12	5.79 ± 0.04	5.76 ± 0.03				
	MyrtM	6.03 ± 0.07	6.01 ± 0.06	5.79 ± 0.01	5.79 ± 0.04				

*Note*: C: ewes from control group fed oat hay plus concentrate; Myrt: ewes fed MDL pellets; MyrtM: leg' meat from Myrt group added with MDL powder; MyrtC: leg' meat from Myrt group; CC: leg meat from C group; CM: leg' meat from C group added with MDL powder; a. b. c: different letters within the same row (different storage days) differ significantly (*p* < .05); MSE: Standard error of the mean.

The use of MDL in ewe's diet had no effect on meat's pH (Table 2). However, the addition of MDL powder had a significant effect (*p* < .05) on overall pH values, where treated groups (MyrtM and CM) presented the lowest values toward the end of the storage period. The storage time significantly (*p* < .05) affected the meat' pH. In fact, the initial pH was similar for all meat samples, ranging between 5.97 and 5.98, then the pH significantly decreased during storage to reach, on the 6th day, the lowest values of 5.79 and 5.76 for MyrtM and CM, respectively.

### 3.3 Lipid oxidation (TBARS) change during refrigerated storage

The results of lipid oxidation are shown in Table 2. The MDL intake had significantly (*p* < .05) decreased the lipid oxidation resulting in lower TBRAS for meat from ewes fed MDL. In addition, the post mortem treatment of deboned leg with MDL powder had a significant effect (*p* < .05) on lipid oxidation of leg meat by lowering TBARS, reaching 0.81 and 0.73 mg MDA/kg of meat, at the end of storage for MyrtM and CM, respectively, while it reached 1.03 mg of MDA/kg for CC group, indicating higher lipid oxidation. The storage time had a significant (*p* < .05) effect on TBARS values of leg meat product. In fact, for all groups, TBARS increased throughout the storage period; and it was with a slower rate for MyrtM and CM than MyrtC and CC.

### 3.4 Microbiological quality of deboned leg during storage

The results of microbiological counts of leg meat samples are reported in Table 3. Neither ewes' MDL intake nor MDL powder addition did affect meat's microbial count.

**TABLE 3:** Effect of distillate myrtle leaves (MDL) addition in pre and postmortem phases on leg meat microbiological quality during storage (Log CFU/g)

	Treatment	Storage days				Statistics				MSE
		0	2	4	6	*p*‐MDL in Diet	*p‐*MDL powder	*p*‐Time	*p* (Time × MDL powder)	
	CC	2.27 ± 0.07	2.6 ± 0.22	4.6 ± 0.17	7.01 ± 0.15					
TVC	MyrtC	3.65 ± 0.04	2.6 ± 0.16	4.1 ± 0.28	7.23 ± 0.10	.4	.18	<.01	.002	
	CM	2.59 ± 0.09	2.4 ± 0.17	4.9 ± 0.21	7.51 ± 0.15					0.07
	MyrtM	3.73 ± 0.06	2.7 ± 0.3	4.0 ± 0.18	7.73 ± 0.10					
	CC	2.62 ± 0.14	2.53 ± 0.25	5.37 ± 0.22	6.85 ± 0.16					
TC	MyrtC	2.84 ± 0.10	2.60 ± 0.31	4.66 ± 0.03	7.34 ± 0.04					
	CM	2.62 ± 0.14	2.45 ± 0.22	5.10 ± 0.16	7.41 ± 0.06	.5	.86	<.01	<.01	0.05
	MyrtM	2.84 ± 0.13	2.05 ± 0.29	4.63 ± 0.11	7.49 ± 0.02					
	CC	1.48 ± 0.09	1.74 ± 0.13	2.13 ± 0.08	3.56 ± 0.17					
FC	MyrtC	1.43 ± 0.08	1.57 ± 0.13	1.54 ± 0.16	3.70 ± 0.01					
	CM	1.48 ± 0.09	1.47 ± 0.12	1.59 ± 0.16	3.46 ± 0.14	.9	.33	<.01	.48	0.04
	MyrtM	1.43 ± 0.08	1.73 ± 0.12	1.51 ± 0.22	3.82 ± 0.10					
	CC	2.12^a^ ± 0.25	2.16 ± 0.06	2.56 ± 0.20	6.58^b^ ± 0.08					
YM	MyrtC	2.28^a^ ± 0.22	2.00 ± 0.24	2.65 ± 0.23	6.76^b^ ± 0.14					
	CM	2.53^a^ ± 0.09	1.93 ± 0.17	2.45 ± 0.17	7.09^b^ ± 0.18	.93	.68	<.01	.8	0.06
	MyrtM	2.74^a^ ± 0.13	2.10 ± 0.10	2.48 ± 0.12	7.17^b^ ± 0.07					

*Note*: C: ewes from control group fed oat hay plus concentrate; Myrt: ewes fed MDL pellets; MyrtM: leg' meat from Myrt group added with MDL powder; MyrtC: leg' meat from Myrt group; CC: leg meat from C group; CM: leg' meat from C group added with MDL powder; TVC: total viable counts; TC: Total coliforms; FC: fecal coliforms; YM: Yeast and mold; a. b. c: different letters within the same row (different storage days) differ significantly (*p* < .05); *p*(time × MDL powder): time and MDL powder interaction; MSE: Standard error of the mean.

The *Clostridium botulinum* and the *Staphylococcus aureus* were not detected throughout the period of storage in all meat samples. However, all microbial counts significantly increased from day 4 of storage (*p* < .05) being inferior to 5.69 log cfu/g at this time, then significantly increased on the 6th day of storage to become higher to 7 log cfu/g for all samples. The total coliforms count remained stable during the first two days of storage than it significantly increased (*p* < .05) to reach, on the 6th day, 7.41 log cfu/g for CM and 6.85 log cfu/g for CC. Fecal coliforms count also presented the same tendency, where it significantly increased on day 6 of storage. During storage, yeast and mold count remains stable until day 4 then increased to exceed 6 log cfu/g for all groups. The overall mean values of TVC and yeast and mold count was higher for MDL powder samples than control ones.

### 3.5 Sensorial analysis

Results of sensorial evaluation are shown in Table 4. Ewe's diet only affected samples' juiciness and aftertaste. In fact, all panel members have detected a ‘sheep’ aftertaste which was stronger in samples from Myrt group. For the juiciness, samples from ewes' fed MDL presented better scores than the control group with mean values of 5.16 and 3.65, respectively. However, the addition of MDL powder significantly decreased scores of juiciness and affected the color score. In fact, the juiciness score of nontreated deboned legs were higher than that of MDL powder enriched deboned legs. Samples from MyrtM group had the highest color score.

**TABLE 4:** Effect of distillate myrtle leaves (MDL) addition in pre and postmortem phases on leg meat sensorial attributes

Diet	Myrt		C		Statistics		
Treatment	MyrtC	MyrtM	CC	CM	*p*‐MDL in diet	*p*‐MDL powder	MSE
Odor	4.57	5.10	5	5.35	.44	.66	.22
Color	4.33^b^	6.23^a^	5.1^ab^	4.95^ab^	.54	.007	.18
Flavor	4.73	5.47	5.35	4.95	.90	.51	.19
Aftertaste	4.67	5.37	4.35	3.55	.048	.11	.26
tenderness	4.80	4.47	3.8	4.00	.10	.40	.23
Slice cohesiveness	5.27	4.93	4.3	4.35	.13	.48	.26
Juiciness	5.25^a^	5.07^ab^	3.7^b^	3.60^b^	<.01	.007	.20
Overall acceptance	5.03	5.43	5.05	4.10	.20	.32	.26

*Note*: C: ewes from control group fed oat hay plus concentrate; Myrt: ewes fed MDL pellets; MyrtM: leg' meat from Myrt group added with MDL powder; MyrtC: leg' meat from Myrt group; CC: leg meat from C group; CM: leg' meat from C group added with MDL powder; a. b. c: different letters within the same row (different storage days) differ significantly (*p* < .05); MSE: Standard error of the mean.

## 4 DISCUSSION

### 4.1 Leg meat's antioxidant activity

The results of ABTS, as indicator of the total anti‐oxidative activity, shown that the use of MDL powder in deboned leg formulation is more efficient than its use in animal diet. The results obtained for TPC and α‐tocopherol content are in agreement with other studies about the effect of rosemary distillated residues intake on meat polyphenol and vitamin E contents (Yagoubi et al., 2021). It is known that meat of grazing cattle and lambs is rich in vitamin E (Lobón et al., 2017); however, meat produced with MDL has greatly higher vitamin E content than that produced by grazing animals (8.4 vs. 3–4 μg/g). These results may be explained by the high TPC and α‐tocopherol content in MDL. Furthermore, the addition of MDL powder to deboned leg had the same tendency as its intake for both diet types (Myrt and C) resulting in α‐tocopherol level for meat from nonconsuming MDL ewes equivalent to that of consuming MDL ewes. In fact, the MDL had the ability to prevent free radical‐mediated oxidation as well as the ability to stabilize radicals created through intramolecular hydrogen‐bonding on further oxidation during the storage period, which attributed to their high phenolic‐concentration (Afoakwah et al., 2015). Similar results were found by Ben Abdelmalek et al. (2018), where rosemary distillation residues intake was proven to improve the oxidative stability of ewes' cooked sausages. They explained their findings by antioxidant power of rosemary and rosemary residues mainly related to their richness in phenolic compounds (Yagoubi et al., 2021).

### 4.2 Deboned leg meat' color parameters and pH changes during refrigerated storage

Results on color parameters confirmed the absence of antioxidant effect of MDL intake. Our findings disagreed other results concerning the change of meat' color parameters in case of rosemary extract intake decrease in a* against an increase in b* (Bañon et al., 2012) and a reverse situation (increase in a* against a decrease in b*) in case of rosemary distillation residues intake (Ben Abdelmalek et al., 2018). However, a* and b* of cooked ham from pigs fed diets enriched with oregano extract or sweet chestnut wood extract were higher than Control during storage (Ranucci et al., 2015). In fact, it is known that meat's color stability and shelf life are associated to oxidation by the action of polyphenols. Thus, ingested polyphenols can act as a natural antioxidant by delaying lipid oxidation, resulting in meat color preservation. However, the use of MDL powder as additive to leg' meat affected a* and b* indices. In fact, the lightness is mostly associated with muscle and protein structures, thus the denaturation of muscle protein might lead to a pale color meat as a variation in muscle structure could affect the reflectance of light scattering (Hayes et al., 2011), which was not the case in the current study. However, a decrease in the luminosity values in Spanish salchichón was recorded when enriched with chestnut leaves at 2 g/kg (Munekata et al., 2017), For redness a*, similarly to our results, Lorenzo et al. (2014) showed that the addition of chestnut leaves extract (1 g/kg) in pork patties resulted in a decrease in redness a* values throughout 20 days of storage. For the current study, the difference of a* indice among groups may be explained by higher polyphenols and tocopherols content in CM and MyrtM meat, which can act as a natural antioxidant (Tibaoui et al., 2020). For b* values, our results are in agreement with Pham et al. (2014) who found that rosemary extract addition in fresh pork sausage increased b* values during 3 months of storage. However, Baldin et al. (2016) showed that sausage of control group presented higher b* than that added by Microencapsulated jabuticaba (*Myrciaria cauliflora*) extract. No significant difference in chroma (C) was found, which indicates the intensity of the color. During storage, a* values decreased. In fact, oxymyoglobin, responsible for the bright red color in meat, is transformed to brown‐colored metmyoglobin; hence, the loss of redness (Renerre, 2000). Phenolic compounds are mostly water‐soluble compounds that would allow direct interaction with myoglobin, a water‐soluble protein. Kroll and Rawel (2001) suggested that this reaction might retard myoglobin oxidation and discoloration. However, McBride et al. (2007), reported a lack of interaction between lipid oxidation and myoglobin oxidation, and showed that phenolic compounds had no effect against meat's color deterioration. Kim et al. (2013) found that beef patties added with plant extract suffered a significant decrease in redness under chilled storage; they explained this result by the greenish color that could be transferred from the vegetable extracts used to the product causing this modification. The yellowness (b*) decreased during storage confirming results of Baldin et al. (2016). Generally, color loss during storage may be attributed to the oxidation process in the presence of oxygen which can be inhibited by the use of natural antioxidants (Falowo et al., 2014).

These results could be explained by to the nature of phenolic compounds contained in MDL powder where the main class is phenolic acids, such as gallic acid, cafeic acid, ferulic acid, and vanillic acid (Aleksic & Knezevic, 2014) contributing to lower pH values. A similar trend was reported by Munekata et al. (2017) where pH decreased with chestnut leaves extracts addition to Spanish salchichón (at concentration of 2 g/kg), they explained these results by chestnut leaves extract high content in acids as gallic, protocatechuic, caffeic, ellagic, ferulic, syringic, and vanillic acid. A reduction in pH was also recorded for sausages added microencapsulated jabuticaba extract (Baldin et al., 2016). However, several studies revealed that plant extract had no effect on meat product pH. Shan et al. (2009) found that the use of five spice and herb extracts as natural preservatives of raw pork had no significant effect on pH among treatments. During storage, pH reductions were probably caused by the increasing lactobacillus count during storage producing lactic acid by break down of carbohydrates (Biswas et al., 2003). These results confirmed the pH decrease when rosemary residues were added to sheep meat (Essid et al., 2018), while Gadekar et al. (2014) recorded no significant difference in goat meat' pH added with natural antioxidant up to 10 days of storage.

### 4.3 Lipid oxidation (TBARS) change during refrigerated storage

Similarly to our findings, it was shown that rosemary residues intake resulted in a significant decrease of TBARS' sausages (Ben Abdelmalek et al., 2018). Likewise, Smeti et al. (2021) have proven the efficiency of myrtle essential oil administration on goat meat against oxidation. The strong antioxidant activity shown by MDL intake and MDL powder treatment is related to their high TPC and α‐tocopherol content. The antioxidant property of polyphenols and α‐tocopherol is associated with their capacity of quenching free radicals; the hydroxyl group linked to the aromatic ring, which is capable of donating hydrogen atoms with electrons and neutralizing free radicals. This mechanism blocks further degradation to more active oxidizing forms, such as malondialdehyde (de Oliveira et al., 2012). As expected, the use of natural antioxidant reduced extent of lipid oxidation. Several authors had reported the meat oxidation decrease by different plant extracts as microencapsulated jabuticaba (*Myrciaria cauliflora*) extract on fresh sausage (Baldin et al., 2016), *fatsia* extract in beef patties' (Kim et al., 2013), extract of cinnamon stick, oregano, clove, pomegranate peel and grape seed in raw pork meat (Shan et al., 2009). Likewise, Hayes et al. (2011) reported the impact of the inclusion of olive leaf extract on the quality and shelf‐life stability of packaged raw minced beef patty. The olive leaf extract was able to inhibit lipid oxidation by reducing TBARS values and significantly reduced oxymyoglobin oxidation in the pack. The significant increase in TBARS values with storage period could be attributed to the production of volatile metabolites in the presence of oxygen (Gadekar et al., 2014).

### 4.4 Microbiological quality of deboned leg during storage

Contrary to our results, several studies have revealed a significant effect of plant distillation extract or residues supply on meat microbiological quality. Thereby, sausages resulting from ewes receiving rosemary residues presented lower TVC than control groups (Ben Abdelmalek et al., 2018). They explained this decrease by the antimicrobial activity of polyphenolic compounds transferred from rosemary diet to sheep meat. It was shown that TVC, psychrophilic count and coliform significantly decreased (*p* < .01) with the addition of rosemary and ginger extracts to lamb patties (Baker et al., 2012), rosemary residues (Essid et al., 2018) or artichokes powder (Afoakwah et al., 2015) to sausage. Authors explained the potential inhibition of microbial growth by the phenolic content that may block adherence and invasion pathogens in the products. In fact, natural plant extracts, including a variety of fruits, vegetables, herbs and spices added to meat and its products have multiple functions. They may play an antimicrobial and preservative role during processing and storage, as plants and plant materials are rich in bioactive compounds. The absence of pathogenic microorganism could be explained by the hygienic practices followed during the preparation of the product object of this study. High microbial counts detected toward the end of storage might be due to the slaughter methods used, a bad preslaughter, carcass and meat handling, or to the equipment and facilities used. In fact, in meat processing, contamination of the product depends on several factors such as the animal, storage conditions, the environment or the personnel involved in the operation (Cerveny et al., 2009).

### 4.5 Sensorial analysis

The addition of MDL powder did delay meat oxidation thus, preventing color deterioration, which explains the high color score allowed to MyrtM samples. Eating quality is a combination of flavor, juiciness, color, and tenderness and it is one of the key aspects that influence consumer's products buying decisions (Grunert et al., 2004). The addition of MDL powder has only affected two of the main components of eating quality (juiciness and color) which may explain the similar acceptability scores among samples. Fernandez‐Lopez et al. (2005) have shown that the addition of rosemary, lemon, and orange extracts in beef meatballs had no negative effect on the acceptability of the product.

## 5 CONCLUSION

In summary, MDL supply in the ewes' diet only affected meat's red color, where Myrt group has an overall higher redness a* values than C group, as well as meat aftertaste and juiciness where samples from ewes' fed with MDL presented better scores than control group. The results also showed that samples treated with M powder presented the highest antioxidant activity and were more stable against lipid oxidation up to 6 days during refrigerated storage, thus, the use of MDL powder retarded the oxidative rancidity. Meat from MyrtM and CM group had higher total phenolic and α‐tocopherol content comparable to control groups. For the microbiological quality, the product was safe up to 4 days of storage and then the microbial count exceeded the recommended limits. The formulation on deboned leg by MDL powder did neither inhibit nor enhance microbial growth. The addition of MDL powder significantly improved color scores, while control samples had better juiciness scores. Such natural extracts contain high levels of bioactive phenolic compounds that can help to control foodborne pathogens and inhibit lipid oxidation; therefore, MDL powder might be used as preservative in meat and for extending shelf‐life. Further studies will be required in order to determine how this powder could be used as health promoting functional ingredients in meat and meat products.

## CONFLICT OF INTEREST

We wish to confirm that the authors report no conflicts of interest associated with this publication.

## ETHICAL APPROVAL

The Animal Care and Use Committee and the Review Board of Animal and Forage Productions Laboratory of the National Institute of Agricultural Research of Tunisia (University of Carthage) considers that the study entitled “Effect of Myrtle leaves integration in sheep diet and its addition as powder on leg meat' oxidative stability, physicochemical, microbiological and sensory properties during storage” falls under the legislation for the protection of animals used for scientific purposes, national decree‐law No. 2005‐95 of 18 October 2005, Chapter II; Sections 1 and 2. It considers that this type of project has no impact on animal welfare because all procedures performed on animals (nutrition and slaughter) are part of routine animal husbandry; the slaughter was carried out according to National regulation and did not involve harm to the animal.

## Data Availability

Research data are not shared.
